# Exploring the Impact of Blood Draws on the Intraocular Pressure of Older Adults: A Focus on Physiological Responses

**DOI:** 10.3390/jcm13216554

**Published:** 2024-10-31

**Authors:** Aida Ramón-Campillo, Inmaculada Bueno-Gimeno, Javier Gene-Morales, Pablo Jiménez-Martínez, Oscar Caballero-Luna, Andrés Gené-Sampedro

**Affiliations:** 1 Department of Optics, Optometry and Vision Sciences, University of Valencia, 46100 Valencia, Spain; airacam@alumni.uv.es (A.R.-C.); inmaculada.bueno@uv.es (I.B.-G.); andres.gene@uv.es (A.G.-S.); 2 Prevention and Health in Exercise and Sport (PHES) Research Group, Department of Physical Education and Sports, University of Valencia, 46010 Valencia, Spain; 3 Research Institute on Traffic and Road Safety (INTRAS), University of Valencia, 46022 Valencia, Spain; 4 ICEN Institute, 28840 Madrid, Spain; 5 Department of Nursing, University of Valencia, 46010 Valencia, Spain; oscar.caballero@uv.es

**Keywords:** blood collection, phlebotomy, venipuncture, glaucoma, ocular health, intraocular pressure

## Abstract

**Background/Objectives**: This study investigated intraocular pressure (IOP) changes after a blood draw in older adults considering sex, age, and baseline IOP. **Methods**: Fifty-three subjects (54.7% females; age: 68.50 ± 4.46 years; Visual Function Index [VF14]: 94.50 ± 7.50 points; mean contrast sensitivity function (CSF) for both eyes in each spatial frequency [cdp]: 1.5 cdp [1.41 ± 0.20 log], 3 cdp [1.57 ± 0.29 log], 6 cdp [1.45 ± 0.39 log], 12 cdp [1.04 ± 0.40 log], 18 cdp [0.63 ± 0.31 log]) voluntarily participated. Subjects fasted for at least 8 h before attending the laboratory. First, IOP was measured in a seated position using a portable rebound tonometer. Ten minutes after the initial measurement, two 10 mL tubes of blood were drawn. Five minutes after the blood draw IOP was measured again following the same procedure as the initial measurement. We evaluated the differences using an analysis of variance. **Results**: Significant, but not clinically relevant, decreases were found in the right eye, with small effect sizes (*p* = 0.013–0.079, d = 0.35). Only males and subjects older than 68 years showed trends toward IOP reduction in the right eye. Subjects with baseline IOP ≥ 14 mmHg experienced significant IOP reductions in both eyes, with moderate effect sizes (*p* = 0.001–0.002, d = 0.56–0.69). **Conclusions**: Our findings suggest that a blood draw of 20 mL is safe for the IOP levels of older adults with baseline IOP between 11 and 21 mmHg. Variations in IOP were observed based on baseline IOP, sex, and age, suggesting the importance of personalized clinical assessments. The primary factor influencing IOP changes appears to be the baseline IOP level.

## 1. Introduction

Intraocular pressure (IOP) is a critical parameter in ocular health, with its regulation being influenced by age and various systemic physiological changes [1,2]. Physiological IOP variations occur in regular rhythmic cycles, and compensatory mechanisms preserve tissue stability [3]. However, when these compensatory mechanisms fail, IOP increases or fluctuations may mechanically compress the optic nerve fiber bundles and cause a discontinuity of axonal transport, therefore, increasing the chances for glaucoma development and progression [3,4]. IOP depends on the balance between aqueous humor production and drain, which is influenced by plasma oncotic pressure, blood pressure in the capillaries, and IOP [5]. In brief, IOP can fluctuate due to different internal and external factors. Internal factors that condition IOP are, among others, systemic blood pressure [6], vascular regulation [7], fluid balance [5], hormonal changes [8], cardiovascular fitness [9], drug intake [10], autonomous nervous system regulation, and psychological factors such as stress [11]. Among external factors, clinical procedures such as blood draws entail physiological changes that may affect IOP [12,13,14].

Blood draws can influence physiological parameters such as blood volume, blood pressure, plasma osmolarity, and fluid balance [12,13,14], and psychological factors such as fear, anxiety, or stress that could influence the autonomous nervous system regulation [15,16,17]. All these physiological and psychological responses potentially derived from a blood draw are associated with the IOP balance [5]. Blood test data were traditionally confined to the clinic for diagnostic purposes but are now becoming more routinely used in many professional and multidisciplinary research settings as a physiological profiling and monitoring tool [18]. Clinical studies and evidence on the specific relationship between blood extraction and IOP are limited, especially in older patients. Therefore, more research is needed to fully understand the relationship between blood extractions and IOP changes. This information is crucial for optimizing patient care in ophthalmology, optometry, and general medical and research practice.

Different responses can be expected according to the blood volume drawn [19]. In this regard, blood donations consist of drawing approximately 10% of the total blood volume (500 mL considering an average of 5 L total blood volume) [20]. On the other hand, between 5 and 50 mL are commonly drawn for clinical routinary extractions [21]. Apart from the volume extracted, blood donors are more accustomed to blood draws and present lesser stress [22]. In adults, a blood donation of 500 mL in patients aged from 20 to 40 years did not affect the IOP 10 min, 1, 2, 3, and 4 h after extraction [19]. Another study [23], with participants aged 21 to 48 years, found no changes in IOP after 30 min of blood donation but observed a significant decrease after 24 h. This reduction in IOP could be due to pressure receptor-mediated sympathetic nervous system activation resulting from reduced blood volume [23]. To the best of our knowledge, no previous research has analyzed the IOP acute responses to a 20 mL blood draw in older adults > 60 years old or considered various factors such as age, sex, and baseline IOP to determine how these variables influence changes in IOP in a short period. Given the limited clinical studies and evidence on this topic, to provide a more comprehensive understanding of this relationship, the question arises as to whether IOP can be influenced by blood draws of volumes commonly extracted for clinical practice in older patients due to physiological or psychological factors.

The aim was to investigate the changes in IOP after a small blood extraction in individuals aged 60 or older, considering the age, sex, and baseline IOP levels. Considering the limited studies consulted, we hypothesized that intraocular pressure would remain unchanged or slightly decrease according to the moderator variables.

## 2. Materials and Methods

### 2.1. Study Design

This was a cross-sectional study with a pre-post within-participant design. The study was conducted under the precepts of the Declaration of Helsinki for experimentation with human subjects and was approved by the Ethics Committee of the University of Valencia (approval: 1861154, date: 5 May 2022). All subjects voluntarily participated in the study and signed an informed consent form. There was no potential risk to participants from blood draws or IOP measurements. Both tests were performed with sterile material on an individual basis, besides IOP measurements and blood draws were performed by trained healthcare professionals. All the measurements were conducted at the Faculty of Nursing of the University of Valencia. The manuscript was prepared according to the STROBE (Strengthening the Reporting of Observational Studies in Epidemiology) guidelines [24].

### 2.2. Participants

We included participants over 60 years of age and leading a sedentary life. We excluded subjects with baseline IOP higher than 21 mmHg, and participants who had undergone refractive surgery and/or glaucoma treatment. Six subjects with these characteristics were excluded to restrict IOP evaluations to a sample without corneal and/or baseline IOP alterations, aiming to find the minimal IOP changes in relatively stable eyes. An a priori sample size analysis (statistical test: matched pairs *t*-tests, two-tailed) with G*Power 3.1.9.6 [25] indicated that 53 subjects were sufficient to obtain a power (1–β) of 0.80, α of 0.50, and a small-moderate effect size of dz = 0.40.

Finally, fifty-three subjects (54.7% females, 45.3% males; age: 68.50 ± 4.46 years; Visual Function Index [VF14]: 94.50 ± 7.50 points; mean contrast sensitivity function [CSF] for both eyes in logarithmic units for each spatial frequency, cycle per degree (cpd): 1.5 cpd [1.41 ± 0.20 log], 3 cpd [1.57 ± 0.29 log], 6 cpd [1.45 ± 0.39 log], 12 cpd [1.04 ± 0.40 log], 18 cpd [0.63 ± 0.31 log]) were selected for final analysis. For the final analysis, participants were divided according to their age group, sex, and baseline IOP levels (see Section 2.5 Statistical Analysis).

### 2.3. Procedures

Participants were scheduled to attend the laboratory between 8:30 and 10:00 am in groups of 5 subjects every 15 min, and they had to fast for at least 8 h. On arrival, each subject rested in a chair for 5 min to equalize baseline conditions. Meanwhile, they answered the VF14, which evaluates self-perceived visual function [26]. CSF was assessed next using the Functional Acuity Contrast Test (FACT) test. Before IOP measurements, subjects were requested to self-report the fear/anxiety derived from a blood extraction on a scale from 0 to 10 points. For the IOP measurement, the patients remained seated with their backs resting against the backrest, looking at a distant target, and both feet on the floor. We first measured the right eye (RE) and then the left eye (LE).

Ten minutes after the IOP measurement, nurses drew two tubes of 10 mL from either arm of the subjects in an adjoining room. Once the withdrawal was completed, they returned to the initial room and sat down for 5 min. After this time, IOP was measured again, following the procedures of the initial measurement.

### 2.4. Variables and Instruments

#### 2.4.1. Visual Function Index

The self-reported visual functional ability was evaluated with the VF-14. This validated questionnaire consists of 14 items enquiring about the visual difficulties perceived during various day-to-day activities (e.g., reading a newspaper, driving, playing sports) [26,27]. The test outcome is scored on a 100-point scale, where 100 is the best visual function and 0 is the worst.

#### 2.4.2. Contrast Sensitivity

The contrast sensitivity was assessed with the FACT test. This test was performed under photopic conditions (>10 lux) monocularly, occluding the non-examined eye. The patients, standing 3 m away from the test, had to indicate the orientation of the sinusoidal pattern with different spatial frequencies measured in cycles per degree (cpd) and contrast. The contrast sensitivity is determined by the last pattern that the subjects discriminate for each spatial frequency and contrast.

#### 2.4.3. Intraocular Pressure

We recorded the average IOP (mmHg) obtained from 6 rapid measurements with an iCare rebound tonometer (iCare100; Tiolat Oy INC, Helsinki, Finland). If large deviations between measurements are detected, the device requires retaking the measurement. Pressures between 11 and 21 mmHg are considered normal in Caucasian subjects, with the average in large cohorts with no ocular signs or symptoms being 15.50 ± 2.75 mmHg [28,29,30]. All the IOP measurements were conducted by the same trained optometrist. The intra-rater test-retest reliability was excellent (right eye: intraclass correlation coefficient [ICC] = 0.952, coefficient of variation [CV] = 1.85%; left eye: ICC = 0.909, CV = 1.87%).

### 2.5. Statistical Analysis

All the statistical procedures were conducted using IBM SPSS (version 28.0, IBM Corp^®^, Armonk, NY, United States), JAMOVI [31], and Orange3 [32]. Data are reported as mean ± standard deviation, and 95% confidence interval (CI). A cut-off criterion of *p* < 0.05 was uniformly established as a criterion for statistical significance.

We conducted, first, basic data curation, codification, and removal of outliers (e.g., IOP > 23 mmHg). Considering data distribution, we used the median to divide the sample into age groups (median: 68 years) and groups formed by the IOP baseline levels (median: 14 mmHg). The normality of data distribution was assessed by the Kolmogorov-Smirnov test. All the dependent variables showed a normal Gaussian distribution (*p* > 0.05) and complied with the sphericity assumption through Mauchly’s W test. Additionally, the intra-rater reliability of IOP measurements was evaluated by performing two measurements on five subjects with five minutes of difference between measurements. For the relative reliability, we calculated the ICC (model 3,1) [33,34], which was interpreted as poor (<0.40), moderate (0.40–0.59), good (0.60–0.79) or excellent (≥0.80) [35]. For absolute reliability, we calculated the CV (standard error of measurement/mean of both measurements) × 100; the standard error of measurement is the standard deviation of the difference between the two measurements divided by the square root of the number of measurements per subject) [36].

Regarding inferential analysis, we conducted, first, a two-way analysis of variance (ANOVA) to assess differences between the pre-and post-extraction IOP from both eyes. The effect size was reported by the eta partial squared (ƞp²), with 0.01 < ƞp² < 0.06 constituting a small effect, 0.06 ≤ ƞp² ≤ 0.14 medium, and ƞp² > 0.14 a large effect. Afterward, post-hoc comparisons were conducted. Considering the small differences, we performed the post-hoc comparisons with three adjustments to visualize the statistical significance at three different levels of robustness (i.e., no adjustment, Tukey, and Bonferroni-Holm). We used Cohen’s d with Hedges’ correction to evaluate the effect size. This parameter was interpreted following Cohen’s guidelines [37] with negligible (d < 0.20), small (d = 0.20–0.40), moderate (d = 0.40–0.80), and large (d > 0.80). Afterward, considering that the differences between eyes were not significant, we conducted two-way ANOVAs to evaluate the potential effects that sex, age, and baseline IOP levels may have on the IOP variations in each eye. The post-hoc analyses for these ANOVAs were conducted with the least significant difference correction (equivalent to no correction) to exhaustively search for any potential difference.

Finally, Pearson’s correlation coefficients (*r*) were graphically calculated for both eyes IOP considering the groups formed according to baseline IOP levels. Similarly, Pearson’s correlations between the fear/anxiety derived from the blood draw and IOP variation in both eyes were calculated to assess psychological influences in IOP.

## 3. Results

The self-reported fear/anxiety derived from the blood draw was 2.5 ± 2.3 points out of a maximum of 10 points. Non-significant correlations were observed between the fear/anxiety and IOP changes in both eyes (*r* between 0.052 and 0.163, all *p* > 0.373).

The ANOVA showed a significant effect of time (F [1, 52] = 5.917, *p* = 0.018, ƞp² = 0.10), but not the eye measured (*p* = 0.215) or the interaction eye*time (*p* = 0.423). While there were no significant differences between eyes (pre-extraction: p_no adjustment_ = 0.500; post-extraction: p_no adjustment_ = 0.086), only the right eye showed a significant but small, not clinically significant decrease in IOP (*p* = 0.013–0.079, *d* = 0.35). The results can be found in Table 1.

### 3.1. Influence of Sex

The effects of time (right eye: F[1, 51] = 8.02, *p* = 0.007, ƞp² = 0.14; left eye: *p* = 0.093) or the interaction time*sex (right eye: F[1, 51] = 4.29, *p* = 0.043, ƞp² = 0.08; left eye: *p* = 0.218) were only significant in the right eye. The post-hoc analysis showed nonsignificant differences or trends between sexes (all p_no adjustment_ ≥ 0.070). However, as can be found in Table 2, only the males showed significant IOP variations or trends (p_no adjustment_ = 0.002 and 0.051).

### 3.2. Influence of Age

Table 3 presents the results according to the age groups formed. There was only a significant effect of time on the right eye (F[1, 51] = 6.29, *p* = 0.015, ƞp² = 0.11). All the rest of the effects and interactions were nonsignificant (all *p* ≥ 0.130). The post-hoc analyses showed nonsignificant differences between age groups (all p_no adjustment_ ≥ 0.209) and significant IOP variations or trends in the subjects ≥ 68 years old (p_no adjustment_ = 0.015 and 0.086).

### 3.3. Influence of Baseline Levels of Intraocular Pressure

The effects of time (right eye: F[1, 51] = 6.36, *p* = 0.015, ƞp² = 0.11; left eye: *p* = 0.013) were only significant in the right eye. On the other hand, the interaction time*sex showed significant effects or trends in both eyes (right eye: F[1, 51] = 3.74, *p* = 0.059, ƞp² = 0.07; left eye: F[1, 51] = 9.94, *p* = 0.003, ƞp² = 0.16). Only the subjects with higher baseline IOP showed significant IOP differences in both eyes (p_no adjustment_ = 0.002 and 0.001). The results are shown in Table 4.

**Figure 1:**
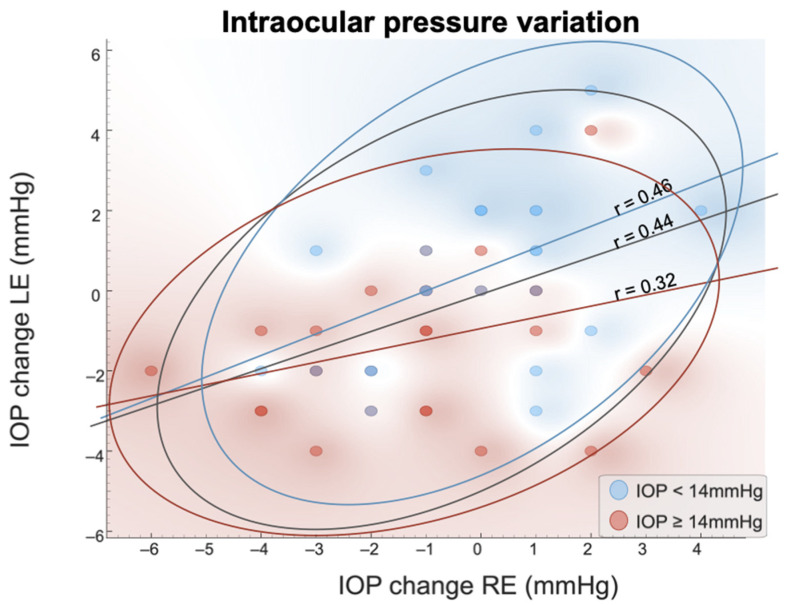
Changes (post–pre) in the intraocular pressure (IOP) of the right (RE) and left eye (LE). Notes for the figure: The colors represent the participants with baseline IOP (average IOP of both eyes) below or above 14 mmHg. Color regions and confidence ellipses were automatically created. The lines represent the correlation between the eyes.

Figure 1 shows the IOP variation (IOPpost–IOPpre) from both eyes dividing subjects according to their IOP baseline levels. Subjects with higher IOP (red region) show a tendency to have their IOP reduced and subjects with lower baseline IOP (blue region) to have their IOP increased. The IOP changes in both eyes were moderately correlated (r = 0.44), being greater in participants with lower baseline IOP (r = 0.46) than in participants with higher IOP (r = 0.32).

## 4. Discussion

To the best of our knowledge, this is the first study to investigate the specific relationship between blood draws and IOP in individuals aged 60 or more, according to their age, sex, and baseline IOP levels. The main finding was that IOP remained unchanged or slightly decreased after the blood draw, the effect sizes for the differences being small to moderate. It is relevant to elaborate on the potential importance of these small changes in a clinical setting, particularly in populations at risk for glaucoma. However, we did not measure glaucoma or hypertensive patients and, therefore, all the implications extrapolated to this population should be taken with caution. Previous research suggests that fluctuations of 1 to 3 mmHg in the long term increase the risk for glaucoma progression [4,38,39,40], and that diurnal IOP variations over 8 mmHg often occur in patients that show glaucoma progression [41]. Therefore, we can consider the average IOP changes in our study, in all cases < 1 mmHg, to be not clinically significant [4]. These findings suggest that a 20 mL blood draw is safe for older adults’ ocular health, reinforcing the idea that blood draws are safe for older adults [20,42].

These findings align with previous studies that observed no significant changes in IOP immediately following blood donation, such as the study by Araz-Ersan et al. [19] which found no significant changes in IOP in adults aged 20 to 40 years before and up to four hours after blood donation. Another study by Yu et al. [23] reported no significant changes in IOP 30 min after blood donation, but a significant reduction in IOP was recorded after 24 h, which was likely due to the activation of the sympathetic nervous system as a result of the diminished blood volume. The slight IOP changes encountered in our study seem to not be modulated by stress/anxiety as the subjects self-reported 2.5 points of stress out of a maximum of 10 points and non-significant correlations were observed.

Although non-significant differences existed between right and left eyes and IOP changes in both eyes were moderately correlated, slightly different IOP behavior was observed in each eye. This highlights the relevance of measuring IOP in both eyes during clinical and research trials, especially considering that IOP asymmetries can be a risk factor for glaucoma development [43]. In the following lines, we discuss the influence of the independent variables selected in this study (sex, age, baseline IOP) on intraocular pressure.

Sex could be a potential factor that conditions intraocular pressure due to sex hormones and genetic variants [44,45]. However, the scientific literature is not consistent, and future research is crucial to better understand the mechanisms underlying sex differences in IOP [45,46]. In this study, although non-significant between-sex differences were encountered, it is worth bearing in mind that males experienced a statistically significant decrease (not clinically significant) in the right eye IOP and a trend toward significance in the left eye, and females did not show significant changes in any of both eyes. These findings contrast with previous research indicating that IOP differences between sexes significantly increase after the age of 40 years [47] and support homogeneous IOP variations between sexes. However, further research is warranted to extract more robust conclusions and unveil potential mechanisms of the differences or similitudes between sexes. Study settings should consider both sexes to ensure robust analyses regarding IOP variations caused by stressors.

The age of subjects seems to play a role in the IOP variation after a blood draw. While subjects younger than 68 years did not show IOP changes, participants aged 68 and older had their right-eye IOP significantly reduced (not clinically significant) and showed a trend in the left eye. This age-related difference might be attributed to changes in vascular and ocular physiology associated with aging, suggesting that older adults might be more susceptible to IOP fluctuations following systemic changes as could be the case of blood draws [48]. Oppositely, it could be hypothesized that younger subjects’ compensatory mechanisms function better to stabilize IOP and avoid significant changes as happened in previous studies [44]. Therefore, caution should be applied when extracting large blood volumes in individuals >70 years, especially for first-time donors [20,42].

The main factor that seems to condition IOP variations with blood draws is baseline IOP levels. This result confirms that IOP levels condition the production and drain of aqueous humor [5] and expands this concept to sustain that IOP changes due to blood draws are also dependent on baseline IOP levels. Subjects with higher baseline IOP experienced significant reductions post-extraction, while those with lower baseline IOP showed slight non-significant increases in the left eye. Therefore, subjects with higher IOP seem to be responders to the physiological stressor (i.e., blood draw), while subjects with lower IOP do not. This could mean that regulatory mechanisms function more optimally in subjects with lower IOP and maintain stable IOP levels after a blood draw. These outcomes are consistent with previous research that encountered larger fluctuations in subjects with higher baseline IOP and highlight the importance of considering baseline IOP in clinical assessments [2,4,44]. Considering these interesting results regarding the different IOP behaviors depending on baseline IOP, future studies should evaluate IOP changes after a blood draw in glaucoma patients with baseline IOP greater than 23 mmHg. These analyses provide the scientific body of knowledge with a new interpretation of how procedures commonly employed in clinical practice and studies such as blood draws can affect older adults’ IOP, providing a foundation to analyze changes according to IOP baseline levels.

All the procedures carried out in this study were carefully designed and supervised, but several limitations and future research directions emerge from the discussion and should be mentioned. First, rebound tonometry was chosen because it is easy to use and does not require anesthesia [49]. However, it does not compensate for corneal thickness. Considering that corneal thickness does not vary with stressors such as exercise [50], this potential bias was diminished by the same experienced optometrist performing all the measurements and, therefore, ensuring within-subject homogeneity. Second, the values presented in this study only reflect pre- and post-blood draw values. Related to this, the use of continuous monitoring devices [51,52] would provide the scientific community with relevant information on what exactly happens during the extraction. Third, future studies should evaluate the effects of blood draws on IOP in older adults from different ethnicities [53] and fitness levels [54] with unadjusted IOP, diagnosed glaucoma, and/or refractive surgery, and characterize subjects according to their refractive status. Fourth, it would be interesting to extend IOP measurements through a larger time frame, e.g., several hours following blood extraction, being able to relate IOP fluctuations to delayed physiological changes inherent to blood draw. In this regard, the assessment of blood pressure and plasma volume pre- and post-extraction could be included aiming to relate changes in blood flow to variations in IOP. Additional variables could be added in future research to increase the scientific contribution to the literature, objectively studying hormones and vasovagal changes related to the stress caused by the blood draw. Finally, comparisons of different volumes of blood drawn may help to elaborate a robust conclusion regarding the safety of blood draws for older patients.

## 5. Conclusions

This study contributes to the limited body of knowledge on the relationship between blood extraction and IOP in older adults. Our findings suggest that a blood draw of 20 mL is safe for the IOP levels of older adults with baseline IOP between 11 and 21 mmHg. However, different IOP behaviors were observed depending on subjects’ baseline IOP, sex, and age, suggesting the importance of personalized clinical assessments. Future studies should further explore these factors and mechanisms involved to optimize patient care in ophthalmology and related fields.

## Figures and Tables

**Table 1:** Intraocular pressure (in mmHg) outcomes.

Eye	Time	Mean	Standard Deviation	Mean Diff. ^a^	95%CI ^b^	Significance Pre-Post			Cohen’s *d*
						No Adjustment	Tukey	Bonferroni-Holm	
Right	Pre	13.83	2.56	−0.72	−0.16–−1.28	0.013 *	0.061	0.079	0.35
	Post	13.11	2.43						
Left	Pre	14.09	3.16	−0.47	−1.07–0.12	0.117	0.391	0.351	0.22
	Post	13.62	2.71						

* Statistically significant differences from pre- to post-blood draw; ^a^: mean difference; ^b^: 95% confidence interval.

**Table 2:** Intraocular pressure (in mmHg) outcomes segmented by sex.

Eye	Sex	Time	Mean	Standard Deviation	Mean Diff. ^a^	95%CI ^b^	Sig. ^c^	Cohen’s *d*
Right	Female	Pre	13.38^(0.160)^	2.41	−0.21	−0.53–0.94	0.574	0.10
		Post	13.17^(0.848)^	2.24				
	Male	Pre	14.96	2.27	−1.33	−2.14–−0.53	0.002 *	0.68
		Post	14.08	2.69				
Left	Female	Pre	13.38^(0.070)^	3.18	−0.14	−0.94–0.66	0.730	0.07
		Post	13.24^(0.265)^	2.87				
	Male	Pre	14.96	2.99	−0.88	−1.75–0.00	0.051	0.39
		Post	14.08	2.48				

* Statistically significant differences from pre- to post-blood draw; ^a^: mean difference; ^b^: 95% confidence interval; ^c^: *p*-value of significance (no adjustment) for the pre-post comparison; the *p*-values of significance (no adjustment) for between-sex comparisons are presented in superscript inside brackets.

**Table 3:** Intraocular pressure (in mmHg) outcomes segmented by age.

Eye	Age	Time	Mean	Standard Deviation	Mean Diff. ^a^	95%CI ^b^	Sig. ^c^	Cohen’s *d*
Right	<68 years	Pre	13.36^(0.209)^	2.66	−0.44	−1.26–0.38	0.285	0.24
		Post	12.92^(0.590)^	2.31				
	≥68 years	Pre	14.25	2.43	−0.96	−1.74–−0.19	0.015 *	0.42
		Post	13.29	2.57				
Left	<68 years	Pre	13.92^(0.709)^	3.33	−0.20	−1.07–0.67	0.645	0.09
		Post	13.72^(0.808)^	3.06				
	≥68 years	Pre	14.25	2.49	−0.71	−1.53–0.10	0.086	0.34
		Post	13.54	2.94				

* Statistically significant differences from pre- to post-blood draw; ^a^: mean difference; ^b^: 95% confidence interval; ^c^: *p*-value of significance (no adjustment) for the pre-post comparison; the *p*-values of significance (no adjustment) for between-group comparisons are presented in superscript inside brackets.

**Table 4:** Intraocular pressure (in mmHg) outcomes segmented by baseline intraocular pressure.

Eye	Baseline IOP	Time	Mean	Standard Deviation	Mean Diff. ^a^	95%CI ^b^	Sig. ^c^	Cohen’s *d*
Right	<14 mmHg	Pre	12.00	2.19	−0.16	−0.96–0.64	0.688	0.08
		Post	11.84	2.56				
	≥14 mmHg	Pre	15.46	2.26	−1.21	−1.97–−0.46	0.002 *	0.56
		Post	14.25	2.36				
Left	<14 mmHg	Pre	11.60	2.60	0.44	−0.36–1.24	0.274	0.20
		Post	12.04	2.45				
	≥14 mmHg	Pre	16.32	1.92	−1.29	−2.04–−0.53	0.001 *	0.69
		Post	15.03	2.15				

* Statistically significant differences from pre- to post-blood draw; ^a^: mean difference; ^b^: 95% confidence interval; ^c^: *p*-value of significance (no adjustment) for the pre-post comparison; the *p*-values of significance (no adjustment) for between-group comparisons were all < 0.001.

## Data Availability

All data underlying this study will be made available upon reasonable request to the corresponding author.
